# Supplementary material for: Coupling effect of key factors on ecosystem services in border areas: a study of the Pu’er region, Southwestern China
Source: PeerJ. 2024 Mar 22;12:e17015. doi: 10.7717/peerj.17015 (PMC10962348; doi:10.7717/peerj.17015)
Supplement: Supplemental Information 1 [file peerj-12-17015-s001.docx]

**Appendix A**

**Table A1**

Validation of each ES and factor. ^a^

| **ES & factors** | **Units** | **Classification** | **Data sources** | **Valuation method** |
| --- | --- | --- | --- | --- |
| FA | t/hm^2^ | Low, 1.00e-5 ~ 1.06e-4;  Medium, 1.06e-4 ~ 6.37e-4;  High, 6.37e-4 ~ 1.20e-3. | Grain output and grain sown area of townships in Pu'er region (2020 Statistical Yearbook of Counties in Pu'er Region). | The method used to calculate **FA** in Guo et al. (2020) was also applied in this study. Spatialize & zonal statistic to the grids used in the study. |
| TR | \ | Low, 0 ~ 0.17;  Medium, 0.17 ~ 0.26;  High, 0.26 ~ 0.47. | Naturalness of vegetation types of Pu'er region (Lin et al., 2016); 2020 road network in Pu'er region (https://www.openstreetmap.org/) | The method used to calculate TR in Jopke et al. (2015) was also applied in this study.  Spatialize & zonal statistic to the grids used in the study. |
| SC | hm^2^/t(time) | Low, 3.73e8 ~ 7.10e8;  Medium, 2.71e8 ~ 3.73e8;  High, 8.17e7 ~ 2.71e8. | Annual precipitation data at 1km resolution (<http://gre.geodata.cn>); soil erodibility factor; 30m resolution DEM data in Pu'er region (http://www.resdc.cn/); crop–management factor; support practice factor (Feng et al., 2021). | The RUSLE model (Ganasri & Ramesh, 2016). was used to estimate the **SC** of Pu'er region. Spatial resolution: 1km. Zonal statistic to the grids used in the study. |
| BD | species number (richness） | Low, 1.76e3 ~ 2.07e4;  Medium, 2.07e4 ~ 3.61e4;  High, 3.61e4 ~ 7.28e4. | 265,876 geographical distribution information of 12593 vascular plants species (GBIF, Global Biodiversity Information Facility; http://www.gbif.org; BIEN, Botanical Information and Ecology Network; https://bien.nceas.ucsb.edu; Chinese Digital Herbarium Database https://www.cvh.ac.cn/; and so on) | The Maxent model (Phillips, Anderson & Schapire, 2006; Sales, Galetti & Pires, 2020; Song et al., 2021) was used to simulate the **species richness** of the vascular plants species in Pu'er region. Spatial resolution: 1km. Zonal statistic to the grids used in the study. |
| INV | \ | Low, 0 ~ 1.42;  Medium, 1.42 ~ 2.83;  High, 2.83 ~ 4.25. | 441 invasive alien species and their location (The List of Alien Invasive species in Yunnan Province (2019 edition)) | The Maxent model was used to simulate the **species richness** of the invasive alien species in Pu'er region to represent the colonization risk. The data was reclassified to 5 grades. Spatial resolution: 1km. Zonal statistic to the grids used in the study. |
| PD | \ | Low, 0 ~ 8;  Medium, 8 ~ 13.33;  High, 13.33 ~ 18. | Landing sites density of the *Spodoptera frugiperda* from Myanmar (Wu et al., 2019) | The data was reclassified to 9 grades to represent the extent of **PD.** Spatial resolution: 0.03°×0.03°. Zonal statistic to the grids used in the study. |
| CI | m | Low, 1.37e5 ~ 2.32e5;  Medium, 6.34e4 ~ 1.37e5;  High, 139.36 ~ 6.34e4. | Vector national boundaries of China, Myanmar, Laos, and Vietnam (http://www.sbsm.gov.cn/). | Euclidean distance from the borderline to the center point of each grid was used to represent the extent of **CI**. The greater the Euclidean distance, the weaker the degree of CI. |
| ET | ton | Low, 0 ~ 2.70e5;  Medium, 2.70e5 ~ 7.62e5;  High, 7.62e5 ~ 2.19e6. | Import and export freight volumes of ports in Pu'er region (China’s Port-of-entry 2019 Yearbook) | The volume of freight traffic (Import & export) was taken as the weighted value of the urban area where each port was located. the rural area was valuated as 0. |
| NDVI | \ | Low, 0 ~ 0.24;  Medium, 0.24 ~0.32;  High, 0.32 ~ 0.47. | Monthly NDVI dataset at 5km resolution (http://www.geodata.cn/). | Direct valuating from the data source. Zonal statistic to the grids used in the study. |
| PRE | mm | Low, 866 ~ 1.02e3;  Medium, 1.02e3 ~ 1.17e3;  High, 1.17e3 ~ 1.31e3. | Annual precipitation data at 1km resolution (http://gre.geodata.cn) | Direct valuating from the data source. Zonal statistic to the grids used in the study. |
| TEM | ℃ | Low, 12.87 ~ 17.72;  Medium, 17.72 ~ 19.77;  High, 19.77 ~ 22.99. | A combined Terra and Aqua MODIS land surface temperature and meteorological station data product in Pu'er region (2003-2017) (https://data.tpdc.ac.cn). | Direct valuating from the data source. Zonal statistic to the grids used in the study. |
| PRO | protected area per grid % | Low, 0 ~ 0,32;  Medium, 0.32 ~ 0.63;  High, 0.63 ~ 0.95. | Vector range of protected area in Pu'er region (Strategic Environmental Assessment Report). | Georeference & zonal statistic to the grids used in the study. |
| SLO | m | Low, 5.53 ~ 13.45;  Medium, 13.45 ~ 21.36;  High, 21.36 ~ 29.27. | 30m resolution DEM data in Pu'er region (<http://www.resdc.cn/>). | Slope calculation & zonal statistic to the grids used in the study. |
| SPI | \ | Mild, -1.10 ~ -0.50;  None, -0.50 ~ 1.42. | Climate Hazards Group InfraRed Precipitation with Station (CHIRPS) precipitation grid point data in Pu'er region (2020) (http://www.chc.ucsb.edu/data/chirps). | Spatial resolution: 0.5°×0.5°. Zonal statistic to the grids used in the study. |
| AP | application load per unit of crop % | Low, 0 ~ 1.36e3;  Medium, 1.36e3 ~ 6.74e3;  High, 6.74e3 ~ 2.03e4. | Application of pesticide, fertilizer and filming, sown area, arable area of townships in Pu'er region（2020 Statistical Yearbook of Counties in Pu'er Region） | The method used to calculate **AP** in Guo et al. (2020) was also applied in this study. Spatialize & zonal statistic to the grids used in the study. |
| AC | \ | Low, 0 ~ 0.33;  Medium, 0.33 ~ 0.67;  High, 0.67 ~ 1. | 2020 road network in Pu'er region ([https://www.openstreetmap.org](https://www.openstreetmap.org/)). | The method used to calculate **AC** in Li et al. (2019) also applied in this study. Spatialize & zonal statistic to the grids used in the study. |
| POP | person | Low, 218.76 ~ 3.18e4;  Medium, 3.18e4 ~ 6.34e4;  High, 6.34e4 ~ 9.50e4. | Population density data at 100m resolution in Pu'er region（<https://www.worldpop.org/>）. | Direct valuating from the data source. Zonal statistic to the grids used in the study. |
| LU | \ | Arable, 1; Forest, 2; Grassland, 3;  Water, 4; Urban, 5. | 30m resolution land use data in Pu'er region (<http://globeland30.org/>). | The land use data was resampled to the grids used in the study. |
| CON | % | Low, 20.01 ~ 39.63;  Medium, 39.63 ~ 59.24;  High, 59.24 ~78.85. | 30m resolution land use data in Pu'er region (<http://globeland30.org/>). | Fragstats 4.2 (Li & Reynolds, 1993) was used to evaluate **CON**. Spatial resolution: 1km. Zonal statistic to the grids used in the study. |

^a^ The classification of SPI was based on the meteorological drought composite index classification table (Wu et al., 2019). The classification of LU was based on the percentage of each category (Arable, Forest, Grassland, Water, Urban) in each grid, and then the grid was assigned the value corresponding to the category (Arable, 1; Forest, 2; Grassland, 3; Water, 4; Urban, 5). with the largest percentage. All other factors were classified by Natural Jenks (Chen et al., 2013).

TR, NDVI, SPI, and AC had no units because they are normalized indices. INV and PD had no units because they reclassified the original graded data, and the numerical size was used to represent the strength of the indicator. LU had no unit because it was just used to characterize the difference in land-use categories.

The corresponding data of each factor were presented in Appendix_B.

**Table A2**

Abbreviation Index

| Full name | Abbreviation | ESs & factors | Abbreviation |
| --- | --- | --- | --- |
| ecosystem service | ES | coupling effect | CE |
| bayesian network | BN | geographic information system | GIS |
| food availability | FA | tourism & recreation | TR |
| soil conservation | SC | biodiversity | BD |
| bio-invasion | INV | transboundary pests & diseases | PD |
| economic transactions | ET | cultural interaction | CI |
| normalized differential vegetation index | NDVI | precipitation | PRE |
| temperature | TEM | protected area coverage | PRO |
| slope | SLO | standardized precipitation index | SPI |
| application of pesticide, fertilizer, and filming | AP | transportation accessibility | AC |
| population density | POP | land use | LU |
| landscape contagion index | CON |  |  |


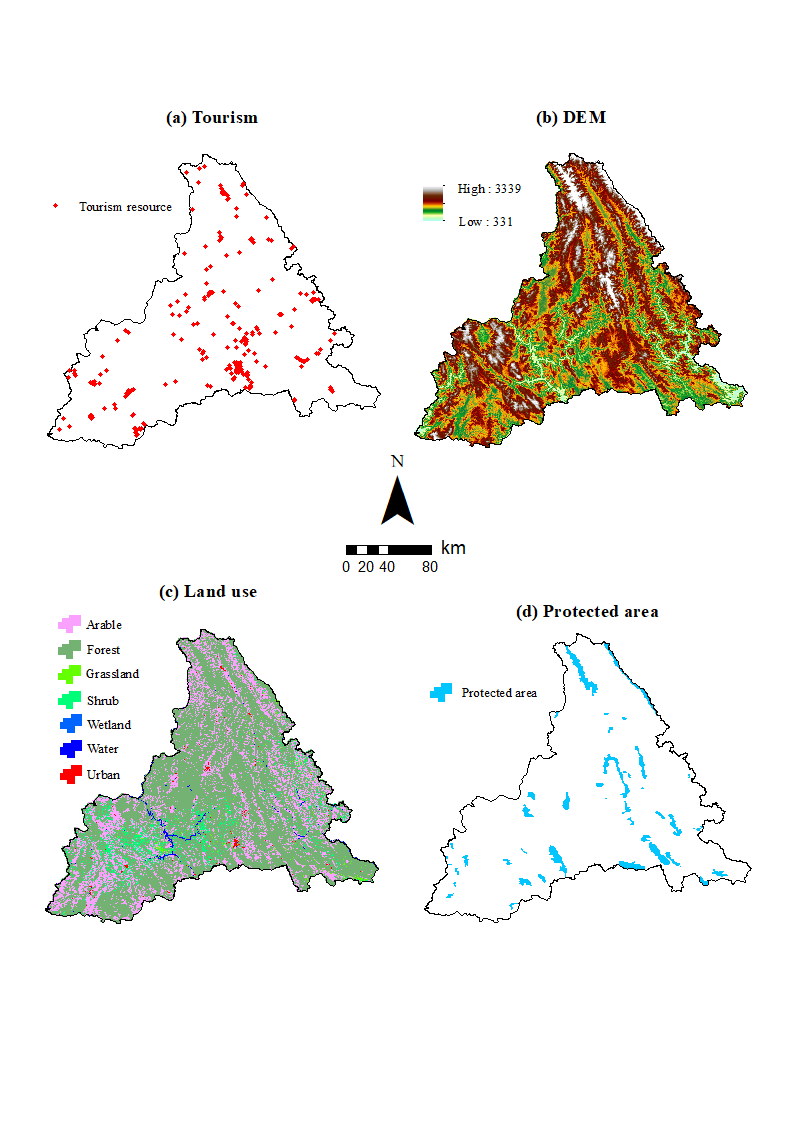


**Fig. A1** Basic geographic information of the Pu'er region. (a) Natural and cultural tourism resource points of the Pu'er region (https://ditu.amap.com/). (b) Topography of the Pu'er region (<http://www.resdc.cn/>). (c) Land use types of the Pu'er region (<http://globeland30.org/>). (d) Protected area of the Pu'er region (Pu'er Forestry and Grassland Bureau).

**Reference**

Chen J, Yang S, Li H, Zhang B, Lv J. 2013. Research on geographical environment unit division based on the method of natural breaks (Jenks). In: *International Archives of the Photogrammetry, Remote Sensing and Spatial Information Sciences - ISPRS Archives*. International Society for Photogrammetry and Remote Sensing, 47–50. DOI: 10.5194/isprsarchives-XL-4-W3-47-2013.

Feng Z, Jin X, Chen T, Wu J. 2021. Understanding trade-offs and synergies of ecosystem services to support the decision-making in the Beijing–Tianjin–Hebei region. *Land Use Policy* 106. DOI: 10.1016/j.landusepol.2021.105446.

Ganasri BP, Ramesh H. 2016. Assessment of soil erosion by RUSLE model using remote sensing and GIS - A case study of Nethravathi Basin. *Geoscience Frontiers* 7:953–961. DOI: https://doi.org/10.1016/j.gsf.2015.10.007.

Guo K, Zhang X, Kuai X, Wu Z, Chen Y, Liu Y. 2020. A spatial bayesian-network approach as a decision-making tool for ecological-risk prevention in land ecosystems. *Ecological Modelling* 419. DOI: 10.1016/j.ecolmodel.2019.108929.

Jopke C, Kreyling J, Maes J, Koellner T. 2015. Interactions among ecosystem services across Europe: Bagplots and cumulative correlation coefficients reveal synergies, trade-offs, and regional patterns. *Ecological Indicators* 49:46–52. DOI: 10.1016/J.ECOLIND.2014.09.037.

Langford E. 2006. Quartiles in elementary statistics. *Journal of Statistics Education* 14. DOI: 10.1080/10691898.2006.11910589.

Li J, Deng W, Zhang JF. 2019. Evaluating mountain water scarcity on the county scale: a case study of Dongchuan District, Kunming, China. *Journal of Mountain Science* 16:744–754. DOI: 10.1007/s11629-018-4964-8.

Li H, Reynolds JF. 1993. A new contagion index to quantify spatial patterns of landscapes. *Landscape ecology* 8:155–162.

Lin SW, Wu RD, Hua CL, Ma JZ, Wang WL, Yang FL, Wang JJ. 2016. Identifying local-scale wilderness for on-ground conservation actions within a global biodiversity hotspot. *Scientific Reports* 6. DOI: 10.1038/srep25898.

Phillips SJ, Anderson RP, Schapire RE. 2006. Maximum entropy modeling of species geographic distributions. *Ecological Modelling* 190:231–259. DOI: https://doi.org/10.1016/j.ecolmodel.2005.03.026.

Sales L, Galetti M, Pires M. 2020. Climate and land-use change will lead to a faunal “savannization” on tropical rainforests. *Global Change Biology* 26. DOI: 10.1111/gcb.15374.

Song HJ, Ordonez A, Svenning J-C, Qian H, Yin X, Mao LF, Deng T, Zhang J. 2021. Regional disparity in extinction risk: Comparison of disjunct plant genera between eastern Asia and eastern North America. *Global Change Biology* 27. DOI: 10.1111/gcb.15525.

Wu Q, Jiang Y, Wu K. 2019. Analysis of migration routes of the fall armyworm Spodoptera frugiperda (J. E. Smith) from Myanmar to China. *Plant Protection* 45:1–6.

Wu W, Li Y, Luo X, Zhang Y, Ji X, Li X. 2019. Performance evaluation of the CHIRPS precipitation dataset and its utility in drought monitoring over Yunnan Province, China. *Geomatics, Natural Hazards and Risk* 10:2145–2162. DOI: 10.1080/19475705.2019.1683082.
