# Supplementary material for: Coupling effect of key factors on ecosystem services in border areas: a study of the Pu’er region, Southwestern China
Source: PeerJ. 2024 Mar 22;12:e17015. doi: 10.7717/peerj.17015 (PMC10962348; doi:10.7717/peerj.17015)
Supplement: Supplemental Information 2 [file peerj-12-17015-s002.docx]

**Appendix B**

*1. Food availability*

The grain output and grain sown area were used to evaluate the FA of the Pu'er region in 2020, FA was calculated by the formula:

(B1)

Where O is the grain output, SA is the grain sown area.

*2. Tourism & recreation*

Tourism and recreation are widely concerned as an important type of cultural services (Millennium Ecosystem Assessment (Program), 2005), which reflects the human pursuit of natural scenery. The nature recreation services depend on the potential capacity of the region to provide natural scenery, and the accessibility of the region (Guo et al., 2020), TR was calculated by the formula:

(B2)

Where N is the naturalness, N is the standardized naturalness (dimensionless), and AC is the standardized transportation accessibility (dimensionless). Referring to the scoring criteria and rules proposed by Lin et al. (2016), and combined with expert knowledge, the naturalness of different vegetation were assigned a value on a 5-point scale, with higher scores indicating higher naturalness of the vegetation type. The value of N was shown in Table 3. The calculation method of TR is explained in detail in Chapter 3.3.7.

*3. Soil conservation*

The USLE model was used to estimate the soil conservation of the Pu'er region in 2020. USLE was calculated by the formula:

${SC}_{i}={RKLS}_{i}-{USLE}_{i}$ (B3)

${RKLS}_{i}=R_{i}K_{i}{LS}_{i}$ (B4)

${USLE}_{i}=R_{i}K_{i}{LS}_{i}C_{i}P_{i}$ (B5)

Where A is the amount of average annual soil erosion; R is the rainfall erosivity factor, K is the soil erodibility factor, LS is the slope length–gradient factor (dimensionless), C is the cover and management factor (dimensionless), which is the ratio of soil loss on a given slope length and slope gradient to soil loss on a typical slope in a standard runoff plot, all other factors being equal. C is the crop–management factor, P is the support practice factor.

In this work, annual scale calculation model was selected to acquire R, and the EPIC model based on soil texture and organic matter content was used to obtain K (Williams, 1990) . The values of C and P combined with the characteristics of the study area and information previous studies are shown in Table 2 (Feng et al., 2021).

**Table B1**

The value of C and P of each land-use type.

| Land use type | Arable | Forest | Grassland | Water | Urban |
| --- | --- | --- | --- | --- | --- |
| C | 0.25 | 0.63 | 0.19 | 0 | 0 |
| P | 0.45 | 0.6 | 0.4 | 0 | 0 |

**Table B2**

Naturalness of vegetation types in the Pu'er region

| Vegetation Types | Naturalness | Vegetation Types | Naturalness |
| --- | --- | --- | --- |
| Dry/hot shrubs | 3 | Semi-humid evergreen broad-leaf forest | 3 |
| Dry land | 2 | Water | 3 |
| Monsoon evergreen broad-leaf forest | 4 | Paddy field | 2 |
| Warm shrubs | 3 | Bamboo forest | 4 |
| Warm coniferous forest | 3 | Warm temperate coniferous forest | 3 |
| Warm-tempered shrubs and thickets | 3 | Tropical and monsoon rainforests | 5 |
| Hot shrubs and thickets | 3 | Mountainous moist evergreen broad-leaved forest | 4 |

*4. Biodiversity*

Maximum Entropy (MaxEnt) is a technique for assessing the probability of distribution based on partial information (Jaynes, 1982). The MaxEnt model was used to estimate the biodiversity of Pu'er Region in 2020. The entropy for the conditional distribution P(Y/X) is:

(B6)

Satisfy the constraint first and then maximize this entropy, the MaxEnt model $P^{*}$ was：

(B7)

*5. Bio-invasion*

The MaxEnt model was used to estimate the bioinvasion of the Pu'er Region in 2020.

*6. Transboundary pests and diseases*

Refer to the data of landing sites and distribution regions of the fall armyworm from Myanmar in spring and summer, after ranking the number of migrations to the Pu'er Region (each grid) in spring and summer, the sum of the number ranks in both seasons was calculated for each grid, which can be used to represent the extent of cross-border pests and diseases.

*7. Cultural interaction*

To represent the extent of cultural interaction, calculate the Euclidean distance from the borderline to the center point of each grid in the Pu'er Region. The greater the Euclidean distance, the weaker the degree of cultural interaction.

*8. Economic transactions*

There are three ports of entry in the Pu'er Region, which are Menglian Port, Mengkang Port and Simao Port, located in Menglian County, Jiangcheng County and Simao District, respectively. We selected the urban area of the Pu'er Region, defined the value of each raster of Menglian County and Jiangcheng County (urban) as the sum of the import and export freight volumes of these two ports. And then, we defined the value of each raster of Simao District (urban) as the sum of value correspondences to Menglian County and Jiangcheng County. The raster values in the Pu'er Region except urban are all defined as 0. The values defined in the above steps are used to represent the extent of economic transactions.

*9. Normalized differential vegetation index*

Direct valuating from the data source. Zonal statistic to the grids used in the study.

*10. Precipitation*

Direct valuating from the data source. Zonal statistic to the grids used in the study.

*11. Temperature*

Direct valuating from the data source. Zonal statistic to the grids used in the study.

*12. protected area coverage*

Direct valuating from the data source. Zonal statistic to the grids used in the study.

*13. Slope*

Direct valuating from the data source. Zonal statistic to the grids used in the study.

*14. Standardized precipitation index*

In this work, standardized precipitation index (SPI) was used to assess drought degree of Pu'er Region in 2020, SPI was calculated by the formula:

(B8)

Where , F is the probability that the random variable X is less than the precipitation in a certain year *X_0_* (Umran Komuscu, 1999). If F > 0.5, F = 1.0 - F; or S = -1.c_0_ = 2.515517, c_1_ = 0.802853, c_2_ = 0.010328, d_1_ = 1.432788, d_2_ =0.189269, d_3_ = 0.001308.

*15. Application* *of pesticide, fertilizer, and filming*

The application of agricultural fertilizers, pesticides, and mulch is the main source of risk for surface source pollution. AP was calculated by the formula:

(B9)

Where P is the application of pollutants by township, S_t_ is the sown area of each township, S_a_ is is the area of the arable in each grid.

*16. Transportation accessibility*

*Transportation* accessibility is the shortest time from each natural village to the administrative center of the county where it is located. The principle of the minimum cumulative resistance (MCR) model is the cost or the work done to overcome the resistance of the landscape with different resistance to get from the "source" to the "destination" (Ye, Yang & Xu, 2020), and the MCR model was used to calculate traffic accessibility:

(B10)


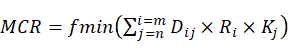


Where MCR is the minimum cumulative resistance value; *f* is an unknown positive function which indicates the positive correlation between ecological suitability and minimum cumulative resistance; min is the minimum value of cumulative resistance taken by a landscape unit to different sources; $D_{ij}$is the spatial distance from source *i* to landscape unit *j*; $R_{i}$ is the resistance coefficient of the landscape unit to the movement process; $K_{j}$ indicates the relative resistance factor of the class to which source *j* belongs; ∑ indicates the calculation of cumulative resistance from plot *i* to source area *j*.

*17. Population density*

Direct valuating from the data source. Zonal statistic to the grids used in the study.

*18. Land use*

The land use types in the Pu'er region in 2020 are defined as different values: arable, 1; forest, 2; grassland, 3; water, 4; and urban, 5. The percentage of each type in each grid is counted, and the grid is assigned to the value corresponding to the land type with the largest percentage.

19. Contagion index

Contagion index reflects the degree of non-randomness or aggregation of different plate types in the landscape (Li & Reynolds, 1993). Fragstats 4.2 was used to evaluate CON. CON was calculated by the formula:

(B11)

Where P_i_ is the percentage of area occupied by type i patches; g_ik_ is the number of type i patches and type k patches adjacent to each other; m is the total number of patch types of the landscape species.

Reference

Feng Z, Jin X, Chen T, Wu J. 2021. Understanding trade-offs and synergies of ecosystem services to support the decision-making in the Beijing–Tianjin–Hebei region. *Land Use Policy* 106. DOI: 10.1016/j.landusepol.2021.105446.

Guo Y, Yang F, Wang J, Wu R. 2020. Assessment of the tourism and recreation cultural ecosystem services in Three Parallel Rivers Region. *Acta Ecologia Sinica* 40:4351–4361.

Jaynes ET. 1982. On the rationale of maximum-entropy methods. *Proceeding of the IEEE* 70:939–952.

Li H, Reynolds JF. 1993. A new contagion index to quantify spatial patterns of landscapes. *Landscape ecology* 8:155–162.

Lin SW, Wu RD, Hua CL, Ma JZ, Wang WL, Yang FL, Wang JJ. 2016. Identifying local-scale wilderness for on-ground conservation actions within a global biodiversity hotspot. *Scientific Reports* 6. DOI: 10.1038/srep25898.

Millennium Ecosystem Assessment (Program). 2005. *Ecosystems and human well-being : synthesis*. Island Press.

Umran Komuscu A. 1999. Using the SPI to analyze spatial and temporal patterns of drought in Turkey. *Drought Network News (1994-2001)*:49.

Williams JR. 1990. The erosion-productivity impact calculator (EPIC) model: a case history. *Philosophical Transactions of the Royal Society of London. Series B: Biological Sciences* 329:421–428.

Ye H, Yang Z, Xu X. 2020. Ecological corridors analysis based on MSPA and MCR model—a case study of the Tomur World Natural Heritage Region. *Sustainability* 12:959.


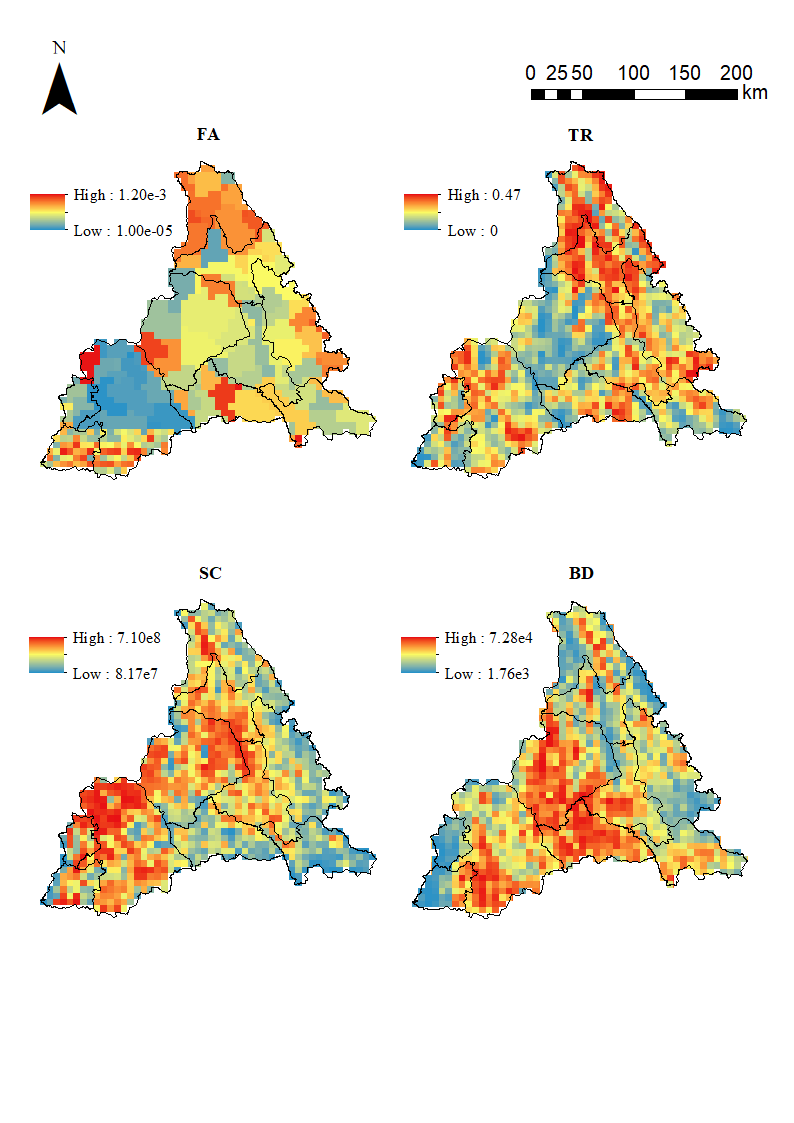


**Fig. B1** Main ESs


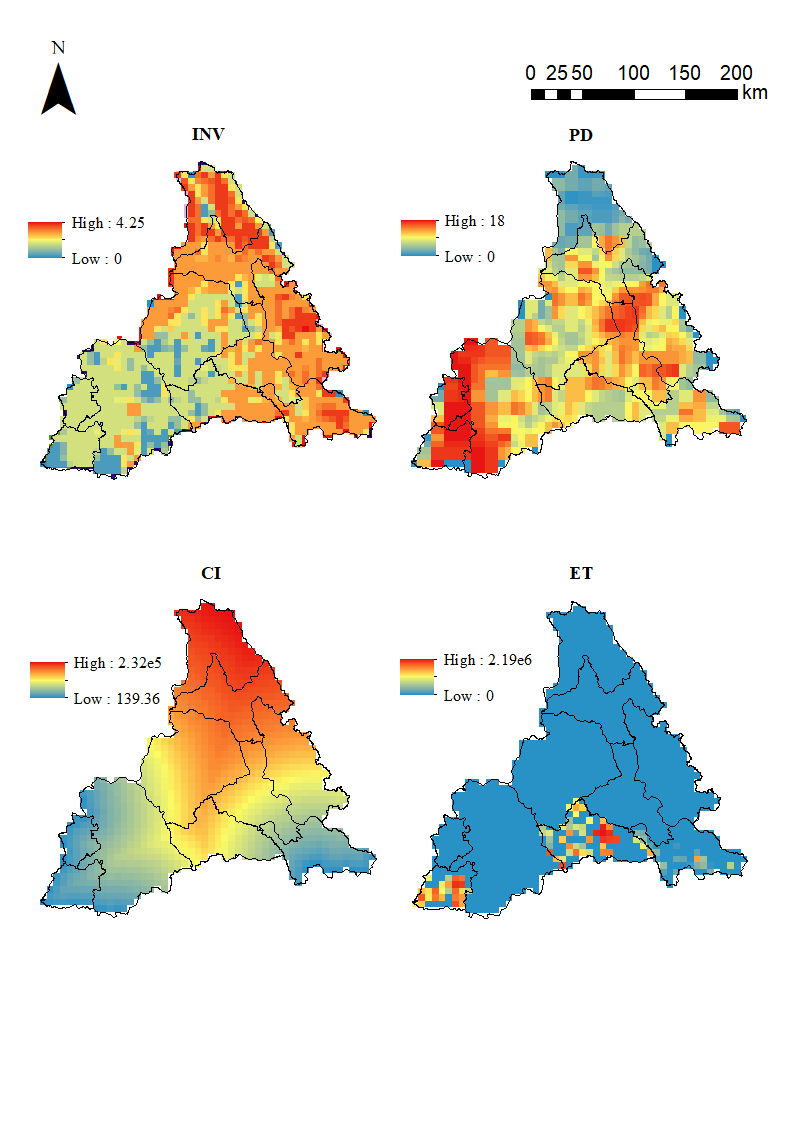


**Fig. B2** Transboundary factors


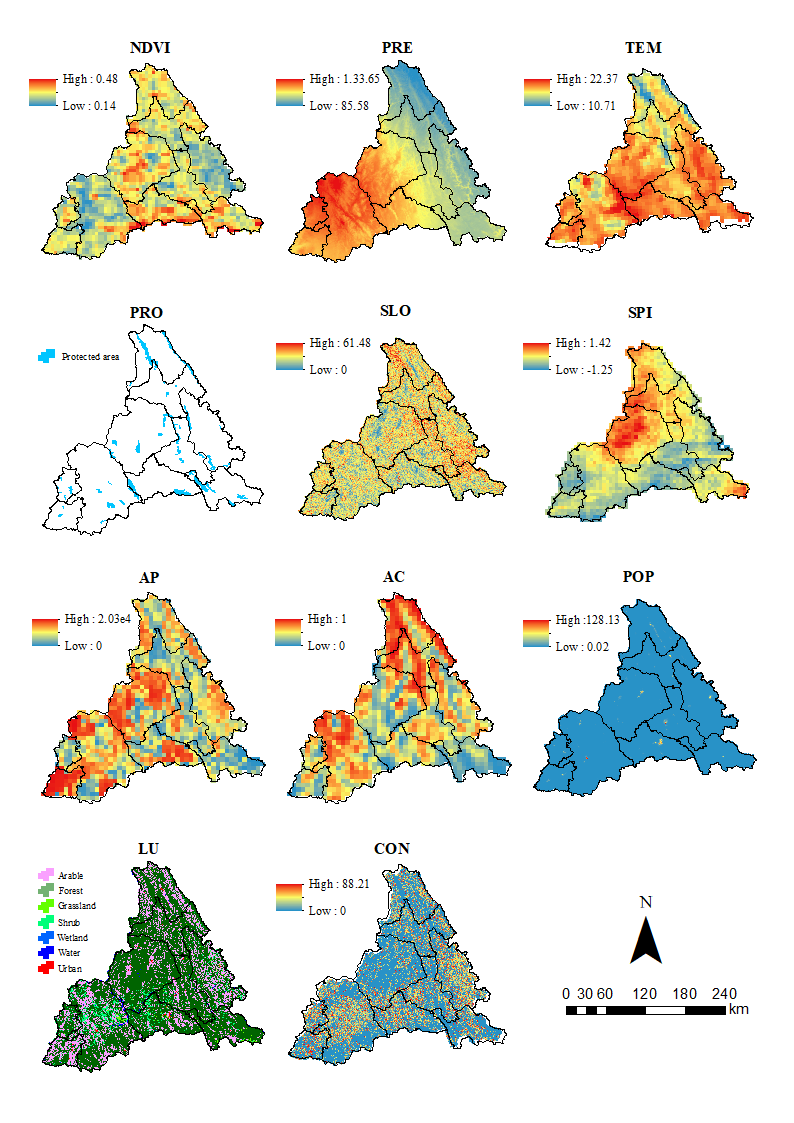


**Fig. B3** Local factors
